# Role of the orexin 2 receptor in palatable-food consumption-associated cardiovascular reactivity in spontaneously hypertensive rats

**DOI:** 10.1038/s41598-018-30970-0

**Published:** 2018-08-23

**Authors:** Shang-Cheng Huang, Tzu-Ling Li, Yen-Hsien Lee, Yu-Wen E. Dai, Yu-Chun Chen, Ling-Ling Hwang

**Affiliations:** 1 Graduate Institute of Medical Sciences, College of Medicine, Taipei Medical University, Taipei, Taiwan; 2 Cheng-Jian Biomedical Company Limited, Taipei, Taiwan; 3 School of Medicine, College of Medicine, Taipei Medical University, Taipei, Taiwan; 4 Department of Physiology, School of Medicine, College of Medicine, Taipei Medical University, Taipei, Taiwan

## Abstract

Hypertensive subjects often exhibit exaggerated cardiovascular reactivity. An overactive orexin system underlies the pathophysiology of hypertension. We examined orexin’s roles in eating-associated cardiovascular reactivity in spontaneously hypertensive rats (SHRs) and Wistar-Kyoto (WKY) rats. Results showed eating regular chow or palatable food (sucrose agar) was accompanied by elevated arterial pressure and heart rate. In both SHRs and WKY rats, the cardiovascular responses associated with sucrose-agar consumption were greater than that with regular-chow consumption. Additionally, SHRs exhibited greater cardiovascular responses than WKY rats did to regular-chow and palatable food consumption. Central orexin 2 receptor (OX2R) blockade attenuated sucrose-agar consumption-associated cardiovascular response only in SHRs. In both SHRs and WKY rats, OX2R blockade did not affect regular-chow consumption-associated cardiovascular responses. Greater numbers of c-Fos-positive cells in the rostral ventrolateral medulla (RVLM) and of c-Fos-positive orexin neurons in the dorsomedial hypothalamus (DMH) were detected in sucrose agar-treated SHRs, compared to regular chow-treated SHRs and to sucrose agar-treated WKY rats. Central OX2R blockade reduced the number of c-Fos-positive cells in the RVLM only in sucrose agar-treated SHRs. We concluded that in SHRs, orexin neurons in the DMH might be overactive during eating palatable food and may further elicit exaggerated cardiovascular responses via an OX2R-RVLM pathway.

## Introduction

Cardiovascular reactivity refers to acute changes in cardiovascular functions in response to physical or mental stimuli or to natural behaviors. Humans and animals with primary hypertension often exhibit exaggerated cardiovascular reactivity, which is suggested to be a predictor or risk factor for the development of primary hypertension^1,2^. Therefore, understanding the mechanisms underlying the exaggerated cardiovascular reactivity is important to provide strategies for the prevention and treatment of hypertension.

In response to daily natural behaviors, blood pressure changes, and a larger increase in blood pressure was observed during eating and drinking than grooming and exploration in rats^3^. The pressor response during eating also exists in humans and many other animals^4–6^. Food presentation and eating normally elicit immediate rises in the arterial pressure, heart rate (HR), and sympathetic activity. These responses last throughout the period of eating and return to basal levels with different recovery kinetics in different species. Eating-associated pressor responses are mainly mediated by central activation of the autonomic nervous system^7–9^.

Previous studies revealed that amplitudes of eating-associated pressor responses vary with the character of the food and with the condition of cardiovascular health as well^10–12^. Consumption of palatable food, compared to non-palatable food, has greater effects on activating the sympathetic vasomotor system in humans^10^. Myers and Scalzo^11^ reported that pups of spontaneously hypertensive rats (SHRs) exhibited a larger increase in blood pressure during feeding than did Wistar-Kyoto (WKY) rat pups. They further found that the exaggerated sympathetic vasomotor activity associated with feeding in SHRs, compared to WKY rats, was responsible for the larger pressor response^12^.

Therefore, activation of the sympathetic vasomotor system is likely an essential factor underlying eating-associated cardiovascular reactivity in both normotensive and hypertensive animals. Nevertheless, the central circuitry responsible for eating-associated elevation of the blood pressure and sympathetic vasomotor activity and for the exaggerated cardiovascular reactivity in hypertensive rats is not fully understood.

The orexin system is known to be involved in regulating feeding and other motivated behaviors, as well as sympathetic vasomotor activity and cardiovascular functions^13,14^. Orexin neurons are activated during eating^15^, and enhanced orexin signals in the ventral tegmental area (VTA) promote reward-related feeding of palatable food^16,17^. In addition, the orexin system is involved in feeding-associated sympathetic activation, as evidenced by the fact that orexin neurons mediate feeding-associated enhancements of glucose utilization in skeletal muscles^18^ and thermogenesis in brown adipose tissues^19^, by increasing sympathetic activity. In summary, orexin neurons are activated during eating, associated with reward-related consumption of palatable food, and involved in feeding-associated sympathetic vasomotor activation. Also, the orexin system is known to promote sympathetic vasomotor activity of the cardiovascular system, thereby elevating blood pressure^20^. We previously demonstrated that elevated orexin 2 receptor (OX2R)-mediated activity contributes to the pathophysiological mechanism of hypertension in SHRs^21^. Therefore, we hypothesized that the orexin system is involved in feeding-associated cardiovascular responses, and elevated orexinergic activity mediates exaggerated cardiovascular responses during palatable-food consumption in SHRs. In the present study, we examined the impacts of food palatability and hypertension on feeding-associated cardiovascular responses and explored the role of the OX2R in responses of SHRs and WKY rats. Cardiovascular responses during eating regular chow or palatable food (sucrose-containing agar) and expression of the Fos protein in orexin neurons and rostral ventrolateral medulla (RVLM) neurons, a critical site in which orexins act to regulate cardiovascular functions, after eating were examined.

## Results

### Eating-associated cardiovascular responses in SHRs and WKY rats

**Figure 1:**
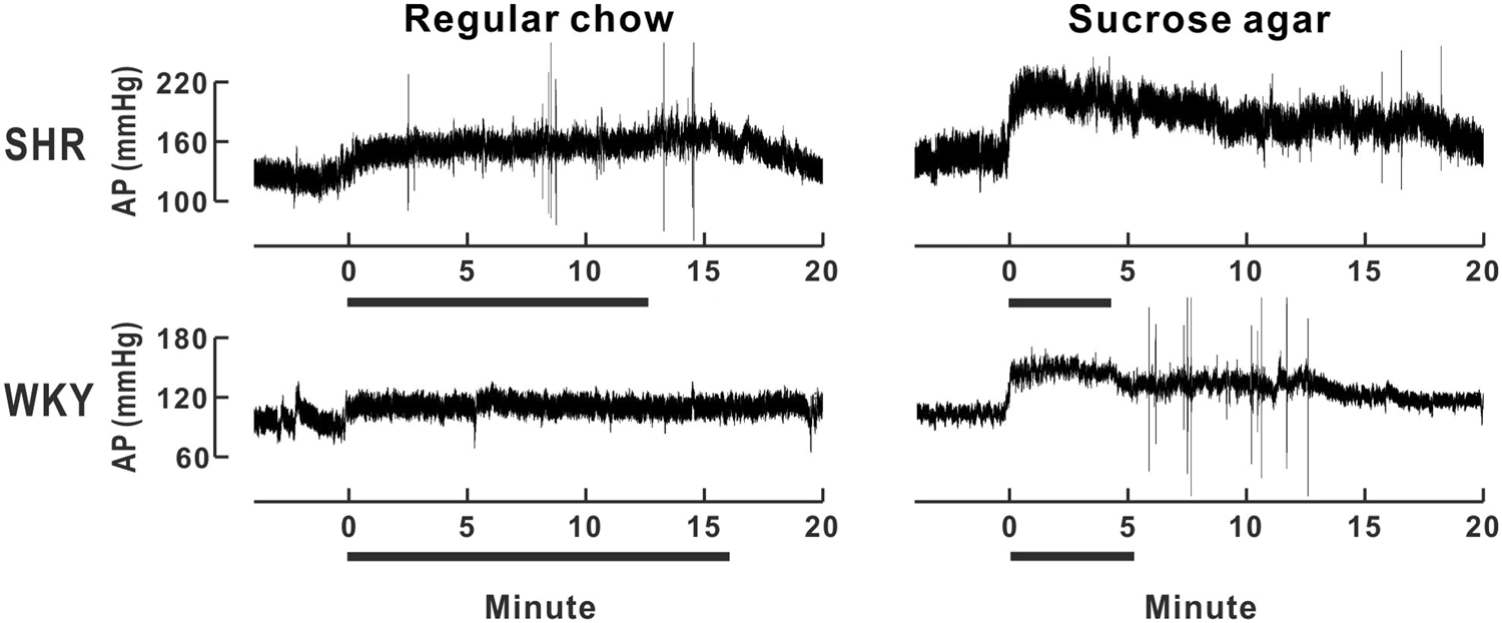
Representative recordings of arterial pressure (AP) from spontaneously hypertensive rats (SHR) and Wistar Kyoto rats (WKY) before, during and after eating either regular chow or sucrose agar. Time zero indicates the time of the rat’s first bite of either the regular chow or sucrose agar and the horizontal lines indicate the duration of food consumption.

After experiencing 12 h fasting, each rat received a treat of 3 g of regular chow or sucrose agar. All animals began to approach and eat the treat within 1 min. Rats completely consumed the sucrose agar in a shorter time (SHRs: 3.6 ± 0.6 min, *n* = 8; WKY rats: 5.7 ± 0.4 min, *n* = 6), compared to the time required to consume the regular chow (SHRs: 12.7 ± 0.7 min; WKY rats: 15.2 ± 1.1 min). SHRs finished consuming the sucrose agar faster than did WKY rats. Representative recording traces of the arterial pressure before and after initiation of eating, as shown in Fig. 1, demonstrated that eating either regular chow or sucrose agar was accompanied by an elevation of arterial pressure, i.e., a pressor response, in both SHRs and WKY rats. The pressor response appeared immediately after initiation of eating, reached its peak within 3 min, and then slowly declined. The pressor response was accompanied by an increase in the HR, which was estimated from the pulse rate of the arterial pressure.

**Figure 2:**
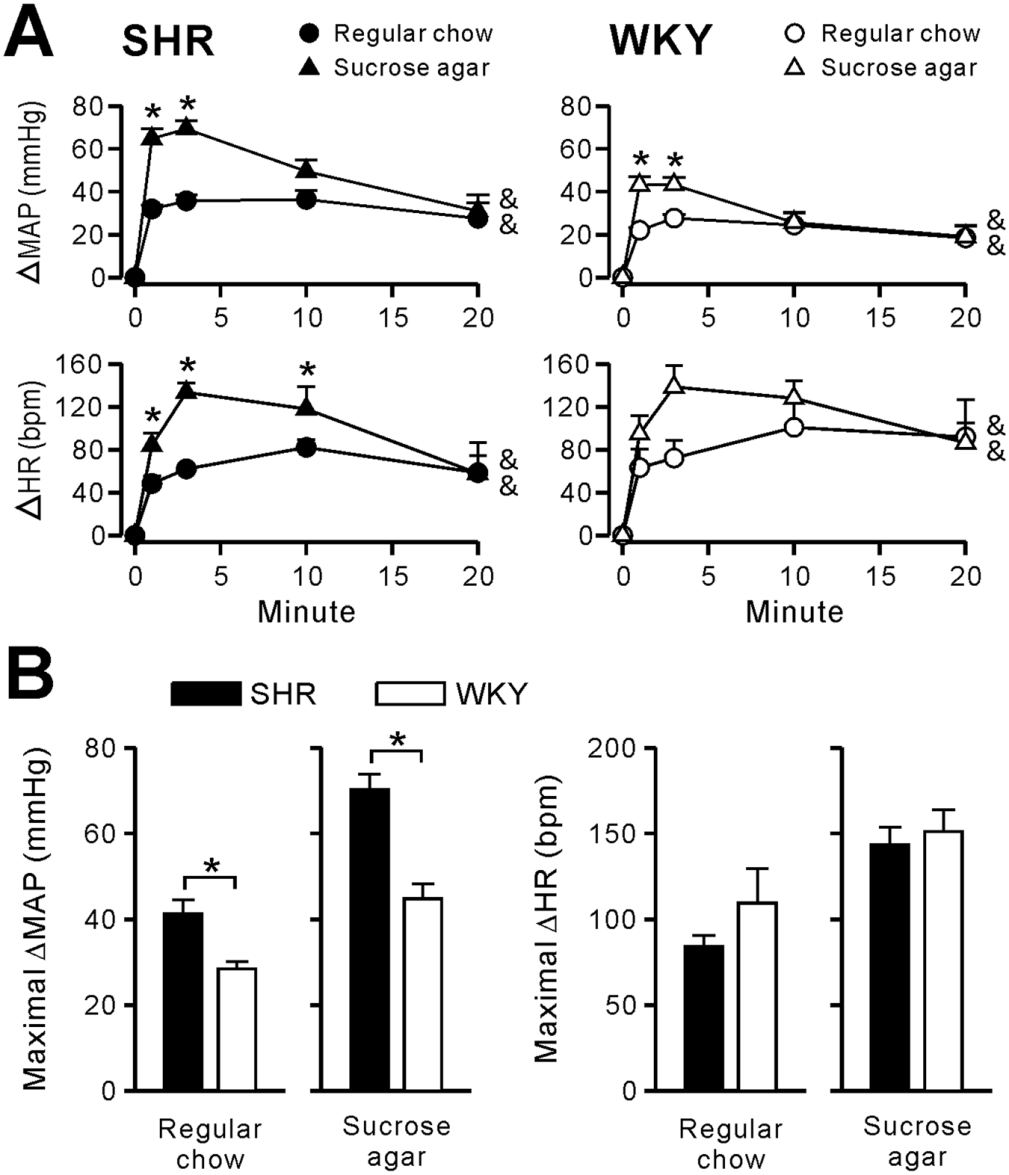
Changes in the mean arterial pressure (MAP) and heart rate (HR) in response to consumption of regular chow or sucrose agar in spontaneously hypertensive rats (SHR) and Wistar Kyoto rats (WKY). Panel A demonstrates the time courses of mean changes in the MAP (ΔMAP) and HR (ΔHR) in SHR (plots on the left) and WKY (plots on the right) after initiating consumption of either regular chow or sucrose agar. An ampersand “&” indicates a significant main effect of each treatment over time on the ΔMAP or ΔHR within each group, as determined by a one-factor repeated-measures ANOVA with the Bonferroni post-hoc correction. There were significant interactions between time and treatment for the ΔMAP in SHR and WKY, and for the ΔHR in SHR. A star “*” indicates a significant difference between regular chow- and sucrose agar-treated groups at the indicated time points, as determined by a post-hoc paired *t*-test. Data are expressed as the mean ± SEM from eight SHR and six WKY. Panel B shows the maximal ΔMAP and maximal ΔHR in response to consumption of either regular chow or sucrose agar in SHR and WKY. Data are expressed as the mean ± SEM from the same experimental groups shown in panel A. A star “*” indicates a significant difference between SHR and WKY, as determined by Student’s *t*-test. bpm, beats per minute.

The change of mean arterial pressure (MAP) and HR (ΔMAP and ΔHR) over 20 min after initiation of eating are shown in Fig. 2A. Eating either regular chow or sucrose agar had significant main effects (indicated with an ampersand “&” in Fig. 2A) on the ΔMAP and ΔHR in both SHRs and WKY rats, as determined by a one-factor repeated-measures ANOVA (SHRs/regular chow/ΔMAP and ΔHR: *F*(4,28) = 17.59, *p* < 0.001 and *F*(4,28) = 14.50, *p* < 0.001; SHRs/sucrose agar/ΔMAP and ΔHR: *F*(1.95,13.65) = 61.53, *p* < 0.001 and *F*(4,28) = 22.78, *p* < 0.001; WKY rats/regular chow/ΔMAP and ΔHR: *F*(4,20) = 12.00, *p* < 0.001 and *F*(4,20) = 5.62, *p* < 0.01; WKY rats/sucrose agar/ΔMAP and ΔHR: *F*(4,20) = 32.24, *p* < 0.001 and *F*(4,20) = 13.74, *p* < 0.001). Therefore, consuming either regular chow or sucrose agar was accompanied by significant elevations in the MAP and HR in both SHRs and WKY rats.

In both SHRs and WKY rats, there were significant interactions between time and treatment for ΔMAP (SHRs: *F*(2.11,29.50) = 9.78, *p* < 0.001; WKY rats: *F*(2.29,22.94) = 4.93, *p* = 0.014). A post-hoc analysis, with paired *t*-test, showed that ΔMAP in response to consumption of sucrose agar was significantly larger than that of regular chow at 1 and 3 min in both SHRs and WKY rats (as shown in Fig. 2A, upper panel). For the time course of ΔHR (Fig. 2A, lower panel), significant interactions between time and treatment were found in SHRs, but not in WKY rats (SHRs: *F*(2.47,34.55) = 4.82, *p* = 0.01; WKY rats: *F*(2.36,23.58) = 1.67, *p* = 0.207). A post-hoc analysis showed that ΔHR in response to consumption of sucrose agar was significantly larger than that of regular chow at 1, 3, and 10 min in SHRs (as shown in Fig. 2A, lower left panel). Therefore, consumption of sucrose agar, compared to regular chow, was accompanied by greater increases in the MAP and HR in SHRs and in the MAP in WKY rats.

We also noted that the amplitude of the responses appeared to differ between SHRs and WKY rats. When consuming either regular chow or sucrose agar, SHRs had a larger maximal ΔMAP than did WKY rats (Fig. 2B, left panel), as determined by an unpaired *t-*test. Maximal values of ΔMAP in response to regular chow were 41.3 ± 3.3 mmHg (*n* = 8) in SHRs and 28.4 ± 1.6 mmHg (*n* = 6) in WKY rats. Maximal values of ΔMAP in response to sucrose agar were 70.2 ± 3.6 mmHg (*n* = 8) in SHRs and 44.8 ± 3.5 mmHg (*n* = 6) in WKY rats. No significant difference was detected in maximal ΔHR values between SHRs and WKY rats that consumed the same treat (Fig. 2B, right panel). Maximal values of ΔHR in response to regular chow were 84.3 ± 6.2 bpm (*n* = 8) in SHRs and 109.5 ± 20.0 bpm (*n* = 6) in WKY rats. Maximal values of ΔHR in response to sucrose agar were 143.5 ± 10.3 bpm (*n* = 8) in SHRs and 151.1 ± 12.6 bpm (*n* = 6) in WKY rats. Therefore, when eating either regular chow or sucrose agar, SHRs exhibited an exaggerated pressor response compared to WKY rats.

### The role of the OX2R in eating-associated cardiovascular responses

Elevated central OX2R activity was proven to contribute to elevated arterial pressure in SHRs^21^. Whether the OX2R is involved in the exaggerated cardiovascular responses in SHRs during eating was examined. An OX2R antagonist, TCS-OX2-29, was intracerebroventricularly (ICV) administered in SHRs and WKY rats to interfere with central OX2R activity.

#### The role of the OX2R in pressor responses to palatable-food consumption

**Figure 3:**
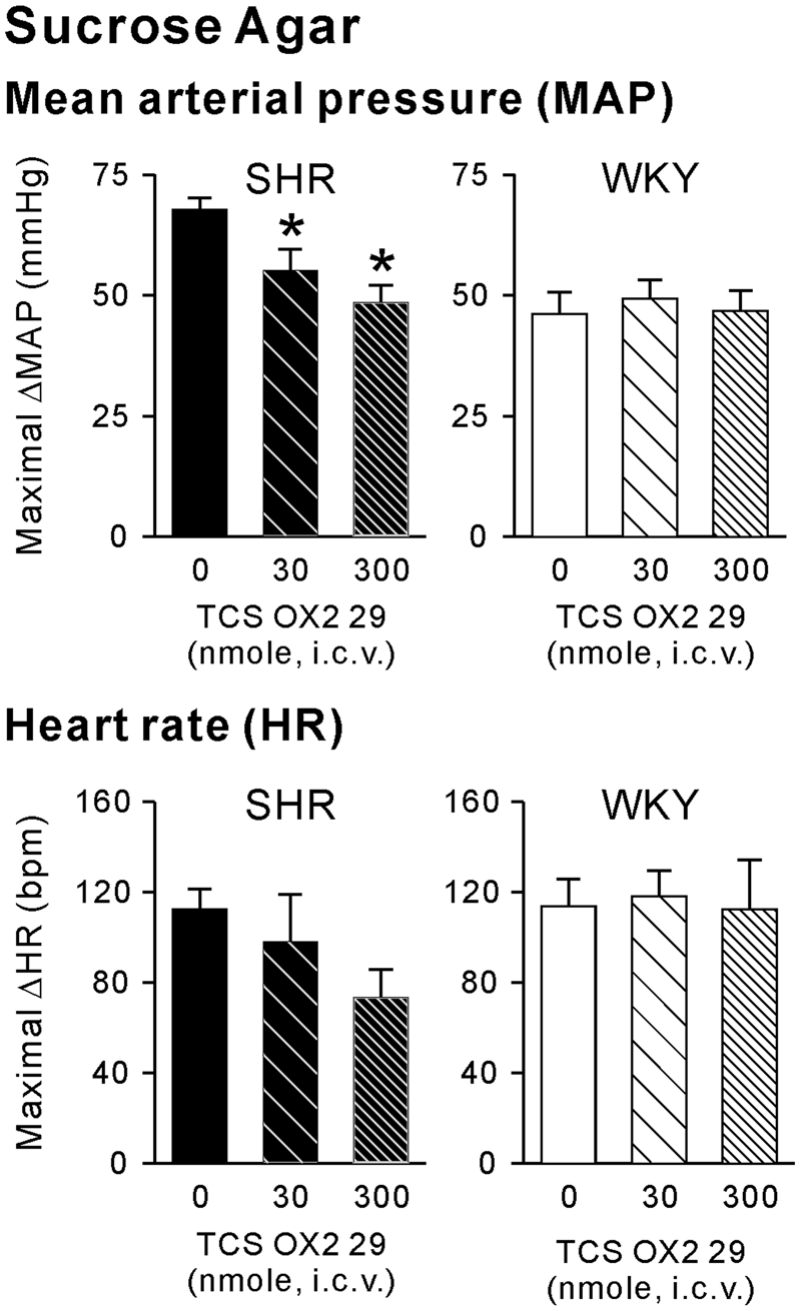
Effects of the orexin 2 receptor (OX2R) antagonist, TCS-OX2-29, on palatable-food consumption-associated cardiovascular responses in spontaneously hypertensive rats (SHR) and Wistar Kyoto rats (WKY). Bar graphs show maximal changes in the mean arterial pressure (maximal ΔMAP) (upper panel) and heart rate (maximal ΔHR) (lower panel) in response to consumption of sucrose agar in SHR and WKY that received intracerebroventricular (i.c.v.) vehicle (n = 6), 30 nmol TCS-OX2-29 (*n* = 6), or 300 nmol TCS-OX2-29 (*n* = 6). Data are expressed as the mean ± SEM. A star “*” indicates a significant difference between the indicated group and vehicle group, as determined by a one-factor ANOVA followed by Duncan’s multiple-range test.

Intracerebroventricularly administrated TCS-OX2-29 at 30 and 300 nmole significantly attenuated the sucrose-agar consumption-induced maximal ΔMAP in SHRs (Fig. 3, upper left panel) but not in WKY rats (Fig. 3, upper right panel), as determined by a one-factor ANOVA followed by Duncan’s multiple-range test. Maximal ΔMAP values of the vehicle, and 30 and 300 nmole groups were 67.7 ± 2.6 (*n* = 6), 55.2 ± 4.3 (*n* = 6), and 48.5 ± 3.6 (*n* = 6) mmHg, respectively, in SHRs, and were 46.2 ± 4.4 (*n* = 6), 49.3 ± 3.8 (*n* = 6), and 46.7 ± 4.3 (*n* = 6) mmHg, respectively, in WKY rats. Maximal ΔMAP values of the 30 and 300 nmole groups in SHRs was not significantly different, suggesting that TCS-OX2-29 at 300 nmole might have nearly reached its maximal effect on the sucrose-agar consumption-induced pressor response. It was noted that TCS-OX2-29 at either 30 or 300 nmole lowered the sucrose-agar consumption-induced maximal ΔMAP in SHRs to levels comparable to those of WKY rats, which was not affected by TCS-OX2-29.

Regarding changes in the HR, the application of TCS-OX2-29 tended to reduce the sucrose-agar consumption-induced maximal ΔHR in SHRs, although it did not reach statistical significance (Fig. 3, lower left panel; 112.3 ± 9.1, 98.3 ± 20.7, and 73.5 ± 12.2 bpm in the vehicle, and 30 and 300 nmole groups, respectively). TCS-OX2-29 had no significant effect on the sucrose-agar consumption-induced maximal ΔHR in WKY rats (Fig. 3, lower right panel; 113.7 ± 12.0, 118.0 ± 11.6, and 112.3 ± 21.9 bpm in the vehicle, and 30 and 300 nmole groups, respectively).

These results suggested that central OX2R activity might not play a significant role in cardiovascular responses elicited by palatable-food consumption in WKY rats. It might only be involved in palatable-food consumption-associated exaggeration of pressor responses in SHRs.

Basal arterial pressures and HRs of the vehicle, and 30 and 300 nmole groups were 142.2 ± 2.6, 140.5 ± 7.3, and 147.2 ± 5.0 mmHg, and 266.0 ± 8.3, 286.7 ± 13.3, and 303.4 ± 12.4 bpm, respectively, in SHRs. In WKY rats, respective values were 97.1 ± 2.2, 84.7 ± 2.7, and 89.6 ± 3.7 mmHg, and 263.9 ± 10.9, 258.8 ± 5.8, and 264.4 ± 12.0 bpm. TCS-OX2-29 at either 30 or 300 nmole had no significant effects on the basal arterial pressure or HR in either strain.

#### The role of the OX2R in pressor responses to regular-chow consumption

**Figure 4:**
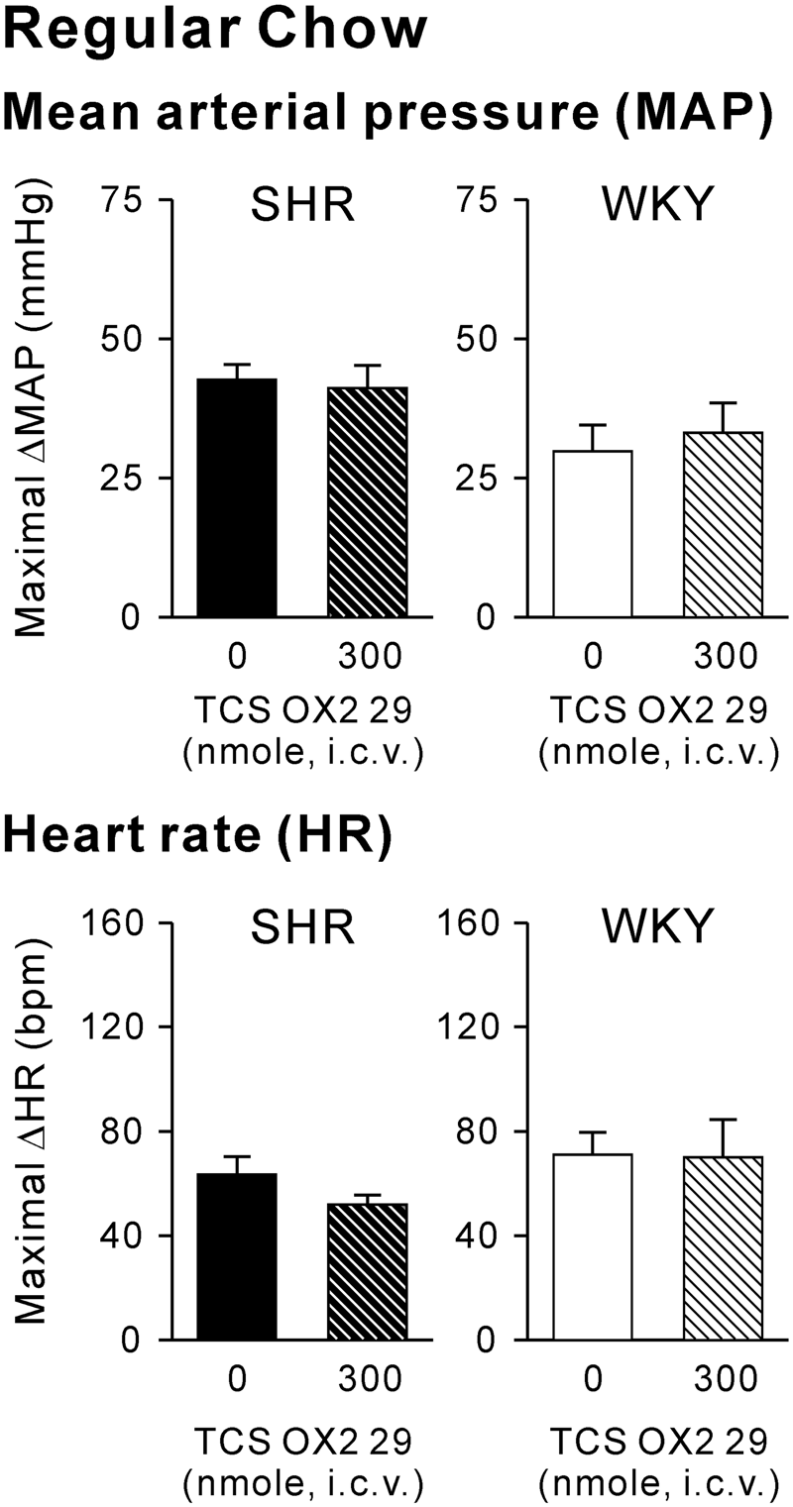
Effects of the orexin 2 receptor (OX2R) antagonist, TCS-OX2-29, on regular-chow consumption-associated cardiovascular responses in spontaneously hypertensive rats (SHR) and Wistar Kyoto rats (WKY). Bar graphs show maximal changes in the mean arterial pressure (maximal ΔMAP) (upper panel) and heart rate (maximal ΔHR) (lower panel) in response to consumption of regular chow in SHR and WKY that received intracerebroventricular (i.c.v.) vehicle (*n* = 6) or 300 nmol TCS-OX2-29 (*n* = 6). Data are expressed as the mean ± SEM.

As mentioned above, SHRs also exhibited exaggerated cardiovascular responses during consumption of regular chow (Fig. 2B). Regular-chow consumption-induced maximal ΔMAP values in either SHRs or WKY rats were not significantly affected by TCS-OX2-29 at 300 nmole (Fig. 4). Values of the maximal ΔMAP were 42.6 ± 2.8 (*n* = 6) and 41.2 ± 4.1 (*n* = 6) mmHg in SHRs and 29.8 ± 4.7 (*n* = 6) and 33.1 ± 5.4 (*n* = 6) mmHg in WKY rats for the vehicle and TCS-OX2-29 groups, respectively. Values of the maximal ΔHR were 63.4 ± 6.9 and 52.0 ± 3.7 bpm in SHRs and 71.0 ± 8.6 and 70.0 ± 14.6 bpm in WKY rats for vehicle and TCS-OX2-29 groups, respectively. Therefore, OX2R activity might not be involved in regular-chow consumption-associated cardiovascular responses in either SHRs or WKY rats, or in regular-chow consumption-associated exaggerated cardiovascular responses in SHRs.

### Expression of c-Fos in the RVLM and roles of the OX2R

SHRs exhibit higher orexinergic activity in the RVLM, and this phenomenon contributes to hypertension in SHRs^22^. Herein, we examined possible involvement of the RVLM in the exaggerated cardiovascular responses associated with palatable-food consumption in SHRs by detecting c-Fos immunoreactivity in the RVLM. SHRs and WKY rats that had received vehicle plus regular chow, vehicle plus sucrose agar, or TCS-OX2-29 (300 nmol) plus sucrose agar were examined.

**Figure 5:**
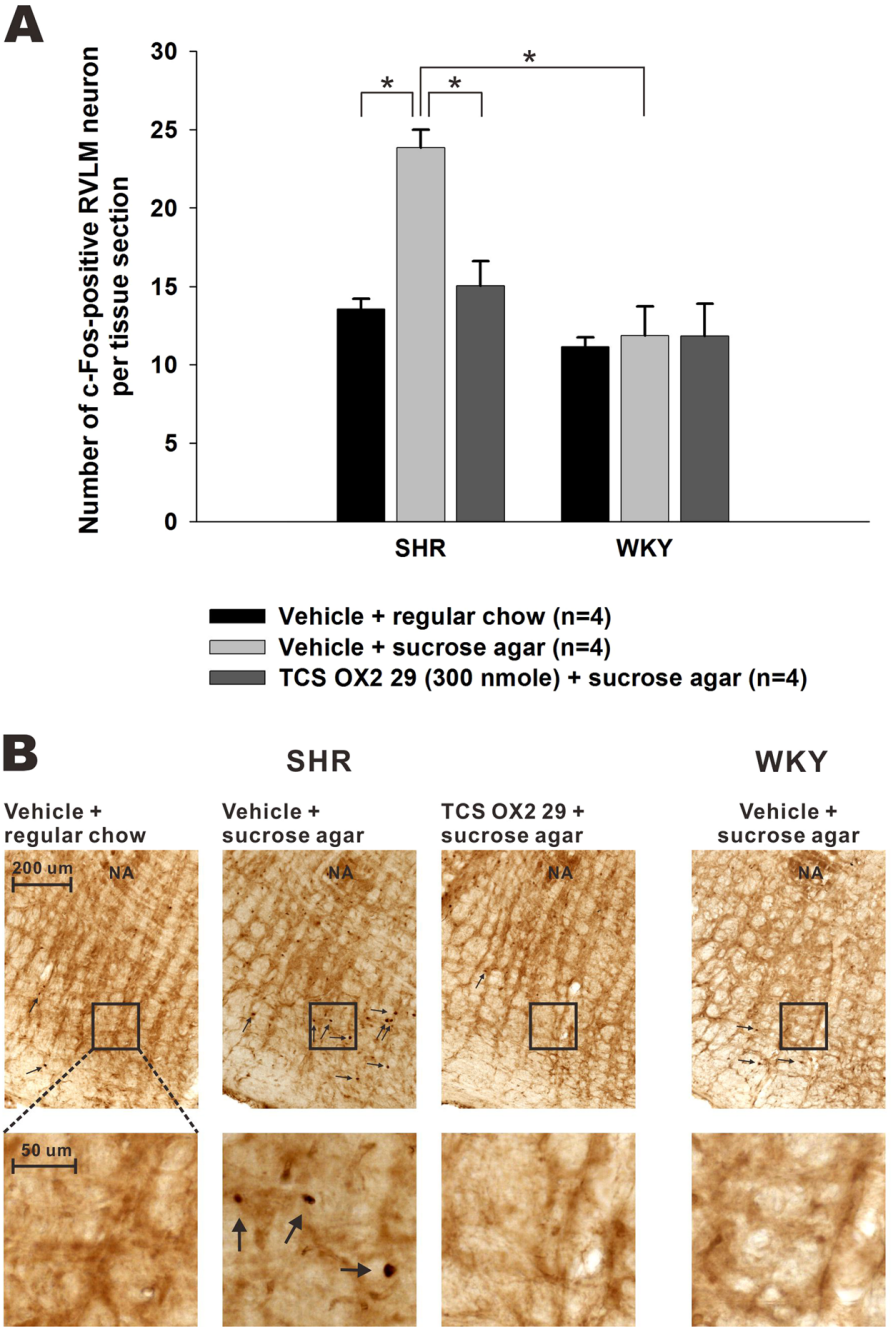
Comparisons of c-Fos expressions in the rostral ventrolateral medulla (RVLM) following food consumption in spontaneously hypertensive rats (SHR) and Wistar-Kyoto rats (WKY) and the roles of the orexin 2 receptor (OX2R). Panel A shows the numbers of c-Fos-immunoreactive RVLM neurons per brainstem slice in SHR and WKY of different treatment groups as indicated. TCS-OX2-29 (300 nmol) or vehicle was intracerebroventricularly applied 30 min before food was presented. Values are the mean ± SEM. A star “*” indicates a significant difference between the indicated groups, as determined with a one-factor ANOVA followed by Duncan’s multiple-range test. Panel B demonstrates photomicrographs of c-Fos-immunoreactivity in the RVLM from SHR that had received treatments as indicated. Arrows indicate cells with c-Fos-immunoreactivity.

Numbers of c-Fos-positive RVLM neurons per tissue section were compared (Fig. 5A). A two-factor ANOVA showed a significant interaction between treatment and species (*F*(2,18) = 6.947, *p* = 0.006), and significant treatment and species effects. Between-treatment comparisons showed that sucrose agar-treated SHRs, compared to regular chow-treated, had more c-Fos-positive neurons in the RVLM (23.9 ± 1.1 and 13.6 ± 0.7 cells/section, *n* = 4), as determined by a one-factor ANOVA with Duncan’s multiple-range test. This difference was only detected in SHRs and not in WKY rats (Fig. 5A), while significant differences in ΔMAP were found in both SHRs and WKY rats (Fig. 2A). Representative images of c-Fos immunostaining are shown in Fig. 5B.

Between-species comparisons showed a significant difference only in sucrose agar-treated groups (sucrose agar: SHRs 23.9 ± 1.1 vs. WKY rats 11.9 ± 1.8 cells/section, *n* = 4; regular chow: SHRs 13.6 ± 0.7 vs. WKY rats 11.2 ± 0.6 cell/section, *n* = 4) (Fig. 5A). It should be pointed out that significant differences in the maximal ΔMAP between SHRs and WKYs were found in both the regular chow- and sucrose agar-treated groups (Fig. 2B). The RVLM is a critical pressor area in the medulla. Results of c-Fos expression in the RVLM suggest that the activity of RVLM neurons might be involved in palatable-food consumption-associated exaggerated cardiovascular responses in SHRs, compared to non-palatable-food consumption and to WKY rats.

Intracerebroventricular application of TCS-OX2-29 (300 nmol), an OX2R antagonist, decreased the number of c-Fos-positive RVLM neurons in sucrose agar-treated SHRs, while it did not affect that in WKY rats (Fig. 5A). Therefore, in SHRs, consumption of palatable food, compared to regular chow, resulted in increased neuronal activity in the RVLM, which may be mediated by activation of the OX2R.

### Expression of c-Fos in orexin neurons

**Figure 6:**
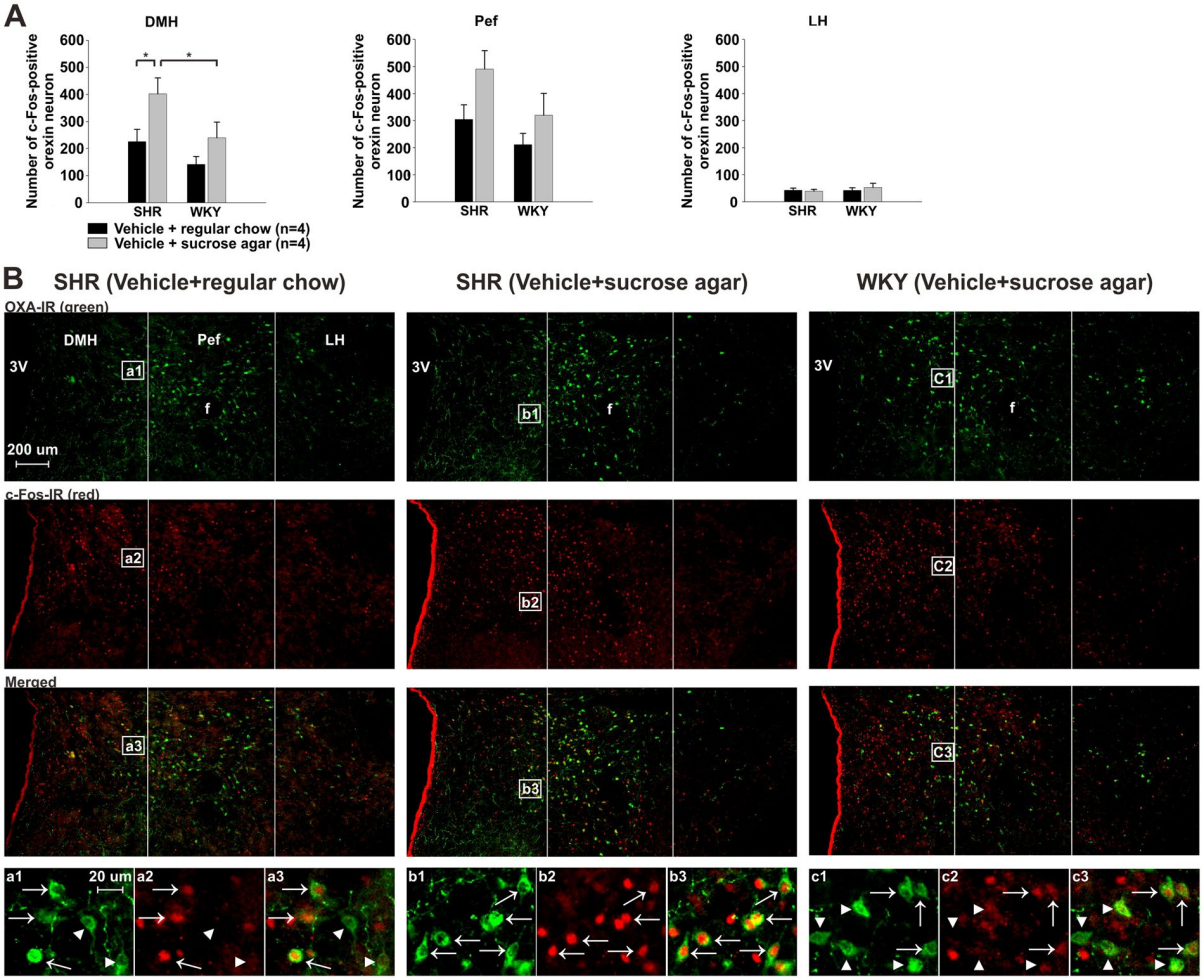
Comparisons of c-Fos expressions in orexin neurons following food consumption in spontaneously hypertensive rats (SHR) and Wistar-Kyoto rats (WKY). Panel A presents the numbers of double-labeled cells in three hypothalamic sub-regions in SHR and WKY of different treatment groups as indicated. Values are the mean ± SEM. A star “*” indicates a significant difference between the indicated groups, as determined by a one-factor ANOVA followed by Duncan’s multiple-range test. Panel B demonstrates fluorescent photomicrographs of the immunoreactivity (IR) of orexin A (OXA) (top row, green), and c-Fos (2nd row, red) in coronal hypothalamic sections (at approximately −3.1 mm to the bregma) from SHR and WKY that had received the indicated treatments. Merged images of OXA- and c-Fos-IR are shown on 3rd row. Bottom row shows images of square regions indicated in three upper rows at higher magnification. The three hypothalamic sub-regions, the dorsomedial hypothalamus (DMH), perifornical hypothalamus (PeF) and lateral hypothalamic area (LH), are divided by two white vertical lines. Arrowheads indicate neurons single-labeled with OXA-IR, and arrows indicate cells double-labeled with OXA- and c-Fos-IR. Abbreviations: 3 V, third ventricle; f, fornix. Scale bars indicate  200 μm in three upper rows and 20 μm in high magnification images (bottom row).

To understand how the activity of orexin neurons were altered in response to consumption of different foods, we compared numbers and percentages of activated orexin neurons in the hypothalamus between regular chow- and sucrose agar-treated rats, and also between species by examining c-Fos immunoreactivity. In addition, it was proven that SHRs have more orexin neurons sending projections to the RVLM than do WKY rats, especially from the DMH and Pef^22^. Therefore, numbers and percentages of c-Fos-immunoreactive orexin neurons were examined in three hypothalamic sub-regions, the DMH, Pef, and LH. Results are shown in Fig. 6A and Table 1.

**Table 1:** Percentages of c-Fos-positive orexin neurons following food consumption in spontaneously hypertensive rats (SHR) and Wistar-Kyoto rats (WKY).

Species/Treatment	Percentage of c-Fos-positive orexin neuron					
	Regular chow			Sucrose agar		
	DMH	Pef	LH	DMH	Pef	LH
SHR (TCS-OX2-29)	57.9 ± 6.5	28.7 ± 3.7	15.2 ± 2.7	74.4 ± 5.1 (70.0 ± 4.9)	41.5 ± 3.7 (36.4 ± 3.4)	13.3 ± 1.6 (12.7 ± 1.8)
WKY (TCS-OX2-29)	41.5 ± 7.1	26.3 ± 1.6	12.8 ± 1.7	58.5 ± 7.9 (38.5 ± 11.2)	38.3 ± 6.7 (24.6 ± 6.3)	17.4 ± 4.2 (9.8 ± 2.5)

Data are expressed as the mean ± SEM (n = 4 in all groups). TCS-OX2-29 treatment was applied in sucrose-agar treated SHR and WKY and the data were shown in brackets. Statistical analysis was performed for each nucleus among experimental groups (not including TCS-OX2-29) with one-factor ANOVA followed by Duncan’s multiple-range test. Effects of TCS-OX2-29 were analyzed with a *t*-test. No significance was found. Abbreviation: DMH, dorsomedial hypothalamus; Pef, perifornical hypothalamus; LH, lateral hypothalamic area.

A two-factor ANOVA revealed no significant interaction between treatment and species in any of the three sub-regions. Significant treatment (*F*(1,12) = 7.883, *p* = 0.016) and species (*F*(1,12) = 6.334, *p* = 0.027) effects were found in the DMH, and a significant treatment effect (*F*(1,12) = 5.526, *p* = 0.037) was found in the Pef. With one-factor ANOVA followed by Duncan’s multiple-range test, sucrose agar-treated SHRs, compared to regular chow-treated SHRs or sucrose agar-treated WKY rats, exhibited more c-Fos-positive orexin neurons only in the DMH (402.3 ± 58.5 vs. 225.5 ± 45.0 and 239.8 ± 57.5, respectively, *n* = 4) (Fig. 6A). A similar trend was noted in percentages of c-Fos-positive orexin neurons in the DMH (74.4 ± 5.1 vs. 57.9 ± 6.5 and 58.5 ± 7.9%), although did not reach statistical significance. Representative images of orexin A- and c-Fos-immunostaining in the hypothalamus are shown in Fig. 6B. It was also noted that a between-treatment difference was not detected in WKY rats, and a between-species difference was not found in regular chow-treated rats in any of the three hypothalamic sub-regions.

In summary, more orexin neuron might have been activated in SHRs that had eaten palatable food, compared to regular chow. This phenomenon was evident in SHRs, but not in WKY rats. Altered orexinergic activation might mainly have occurred in the DMH.

In addition, the effect of TCS-OX2-29 in suppressing c-Fos expression was observed only in the RVLM (Fig. 5), but not in orexin neurons in either sucrose agar-treated SHRs or WKY rats (SHRs: 402.3 ± 58.5 cells in the vehicle plus agar group vs. 366.3 ± 33.5 cells in the TCS-OX2-29 plus agar group; WKY rats: 239.8 ± 57.5 cells in the vehicle plus agar group vs. 190.3 ± 69.0 cells in the TCS-OX2-29 plus agar group; *n* = 4 for all groups) (not shown in Fig. 6). Likewise, percentages of c-Fos-positive orexin neuron between vehicle and TCS-OX2-29 treated groups were not significantly different in either sucrose agar-treated SHRs or WKY rats (Table 1). These findings raise a possibility of that the OX2R might play a minor role in palatable-food consumption-associated activation of orexin neurons.

Control experiments were carried out in two groups of SHRs that went through all procedures, except food presentation, with and without TCS-OX2-29 treatment. Numbers of c-Fos-positive orexin neurons in three hypothalamic sub-regions were not significantly different between the two groups with and without TCS-OX2-29 treatment (with vs. without TCS-OX2-29 in the DMH: 289.0 ± 6.5 vs. 249.3 ± 55.1; in the Pef: 376.3 ± 59.7 vs. 309.3 ± 30.9; in the LH: 35.3 ± 10.2 vs. 44.3 ± 7.7, n = 3).

## Discussion

The present study demonstrated the following main findings: (1) consumption of palatable food, compared to regular chow, was accompanied by greater cardiovascular responses in both SHRs and WKY rats; (2) when eating either regular chow or palatable food, SHRs exhibited exaggerated cardiovascular responses compared to those of WKY rats; (3) while not playing a significant role in cardiovascular responses elicited by eating palatable food in WKY rats, central OX2R activity may be involved in the palatable-food consumption-associated exaggerated cardiovascular responses in SHRs; (4) in either SHRs or WKY rats, central OX2R activity might not contribute to regular-chow consumption-associated cardiovascular responses, or to the exaggerated cardiovascular responses associated with regular chow intake in SHRs; (5) in SHRs, but not in WKY rats, consumption of palatable food, compared to regular chow, resulted in increased neuronal activity in the RVLM, which may be mediated by the OX2R; and (6) in SHRs, consumption of palatable food, compared to regular chow, resulted in a greater number of activated orexin neurons in the DMH. This phenomenon was not observed in WKY rats. Therefore, we propose that eating palatable food, compared to low-reward/low-palatability food, may result in activation of a greater number of orexin neurons in the DMH in SHRs, but not in WKY rats. This may lead to elevated orexinergic activity onto the RVLM via the OX2R, which may underlie palatable-food consumption-associated exaggerated cardiovascular responses in SHRs.

In rats, eating is usually associated with a considerable increase in blood pressure^3^, which is mediated by sympathetic vasomotor activation^7^. Herein, we demonstrate that food palatability affected the magnitude of eating-associated cardiovascular reactivity in rats; consumption of palatable (sweet) food caused greater responses. This phenomenon had not previously been established in rats. This observation is important because it resembles responses observed in humans of ingestion of highly palatable food having a greater effect on activating the sympathetic vasomotor system compared to non-palatable food^10^.

The central orexin system plays important roles in regulating sympathetic vasomotor activity and cardiovascular functions, as well as in the pathophysiology of hypertension^23^. An overactive orexin system contributes to elevated blood pressure in various hypertensive models of rodents, including stress-induced hypertensive rats^24^, SHRs^21,25^, and BPH/2 J mice^26^. In the present study, we discovered a new role of the orexin system in the phasic control of cardiovascular homeostasis associated with palatable-food consumption in SHRs.

The involvement of the orexin system in psychological stress-associated cardiovascular responses is well established^23^. This was first demonstrated by cardiovascular responses to a social stress (the resident-intruder paradigm), but not to tail pinching, being attenuated in orexin-knockout mice^27^. Furlong *et al*. confirmed this notion in wild-type rats by examining various forms of arousal and stress and detecting Fos expression in orexin neurons, orexin levels in the CSF, and the effect of a systemically applied almorexant (an antagonist of the orexin 1 receptor (OX1R) and OX2R) on associated cardiovascular responses^28^. They demonstrated that activation of orexin neurons contributes to cardiovascular responses to forms of stress or arousal that are associated with a high level of attention to the surrounding environment, such as exploration (novelty) and conditioned fear. The contribution of orexin was not seen in the cardiovascular response to restraint stress or cold exposure. Involvement of the OX1R and OX2R is not fully understood and may vary depending on the type of stressor^23^.

In hypertensive animals, cardiovascular responses to stressors are often exaggerated. The role of orexin in phasic regulation of cardiovascular functions in hypertensive animals was examined in two recent studies. In Martin *et al*.’s study^29^, an air-jet stress elicited pressor and tachycardia responses in both SHRs and Wistar rats, but, responses were greater in SHRs. They demonstrated that a systemically applied almorexant at 30 mg/kg caused a greater reduction in the air-jet stress-induced pressor response in SHRs than in Wistar rats (approximately a 65% vs. a 33% reduction). At a higher dose of 100 mg/kg, almorexant reduced the pressor response to similar extents (approximately 50%) in both strains. Systemic administration of a selective OX1R antagonist had no significant effect on the pressor response to an air-jet stress in either strain. These observations suggested a significant role of orexin in cardiovascular responses to acute stress in both hypertensive and normotensive rats. It seems that orexin is involved in exaggerated pressor responses to acute stress in SHRs, which may mainly be mediated by the OX2R.

Another study by Li *et al*.^30^ examined the role of orexin in an augmented chemoreflex in SHRs. Chemoreflexes can be elicited by activation of central or peripheral chemoreceptors. Activation of central chemoreceptors by hypercapnia leads to increases in sympathetic vasomotor activity, blood pressure, HR, and the rate and depth of respiration. These responses are exaggerated in SHRs. Li *et al*.^30^ found that hypercapnia stress activated more orexin neurons in both Pef and DMH and with higher expression in Pef area of adult SHRs. Blockade of orexin receptors with almorexant normalized the augmented hypercapnia-elicited chemoreflex in SHRs, suggesting a critical role of orexin in the augmented hypercapnia-elicited chemoreflex.

Our findings suggest that activation of DMH orexin neurons plays a significant role in palatable-food consumption-associated exaggerated pressor response. The OX2R is likely the major receptor type responsible for this. Our results together with those of the above-mentioned studies suggest that orexin is likely a critical mechanism underlying exaggerated cardiovascular responses to various forms of stressors or behavioral challenges. Therefore, orexin could become a therapeutic target for reducing exaggerated cardiovascular responses in prehypertensive or hypertensive conditions.

The RVLM is an important pressor area in the brainstem. It contains premotor neurons that project to sympathetic preganglionic neurons in the thoracic spinal cord that innervate the cardiovascular system. The RVLM is a critical action site for overactive orexin signaling in regulating cardiovascular functions in SHRs. Overactivity of the orexin system which underlies the pathophysiology of hypertension in SHRs was first demonstrated by two previous studies^21,25^. Clifford *et al*. and Lee *et al*. reported that SHRs have greater numbers of orexin neurons in the DMH and Pef than WKY rats^22,31^. Lee *et al*.^22^ further demonstrated that SHRs have a greater number of orexin neurons that send projections to the RVLM than do WKY rats, especially from the DMH and Pef, and also have augmented OX2R signaling in the RVLM. Our findings of overactive orexin neurons in the DMH and involvement of OX2R in the activation of RVLM neurons are in agreement with those previous findings. An OX2R-RVLM pathway may be important in mediating cardiovascular responses downstream of overactive orexin signaling in SHRs. Whether c-Fos-positive RVLM neurons are responsible for cardiovascular responses observed in the present study requires further study.

In the present study, ICV application of TCS-OX2-29 at either 30 or 300 nmole had no effects on resting arterial pressure and HR in SHRs and WKY rats. This differs from our previous findings that ICV TCS-OX2-29 at 10 and 30 nmole caused significant reductions in the arterial pressure and HR in SHRs. The present study was done on conscious rats, whereas the previous study used anesthetized rats. Different levels of sympathetic vasomotor activity and orexinergic activity in conscious versus anesthetized rats are a possible reason for this difference. A higher dose may be required to suppress OX2R activity in conscious rats. Therefore, the role of RVLM-OX2R in the cardiovascular responses observed in the present study remains to be determined.

Orexin neurons are sensitive to signals of the metabolic status^32–36^. Activation of orexin neurons by signals of energy deficit may promote food-motivated behaviors, via downstream actions on the VTA^17,36,37^. In addition, the OX2R is involved in regulating wakefulness^38,39^. Whether the suppression of palatable-food consumption-associated cardiovascular responses in SHRs by TCS-OX2-29, an OX2R antagonist, resulted from alterations of feeding motivation or wakefulness should be concerned. However, TCS-OX2-29 (300 nmol)-treated SHRs completely consumed the sucrose agar in 3.6 ± 0.5 min (*n* = 6), which did not significantly differ from the time that vehicle-treated SHRs spent, 3.9 ± 0.5 min (*n* = 6). In addition, rewards are most closely associated with OX1R activation^40–42^. Further, Sheng *et al*.^36^ demonstrated that the orexin neuron-VTA pathway that relays metabolic state to reward-based feeding is mediated by the OX1R.

Accumulating evidence demonstrates altered activities of orexin neurons in response to feeding-associated behaviors in both appetitive^40,43^ and consummatory aspects^15,44^. Harris *et al*.^40^ reported that increased c-Fos expression of LH orexin neurons is linked to food reward-seeking behaviors. Choi *et al*.^43^ demonstrated that context-dependent expectations of either a daily meal or a palatable food reward increased c-Fos expression in orexin neurons in the Pef, and central administration of orexin A increased the motivation to obtain the food reward. Mileykovskiy *et al*.^15^ demonstrated that activities of the orexin neurons that project to the VTA or locus coeruleus (LC) increased upon the animal being presented with novel food, decreased as rats explored the food, and increased during consumption of the food, as determined using unit recordings in freely moving rats. Recently, González and colleagues^44^ demonstrated that the activity of orexin neurons decreased immediately after eating onset and remained low during eating, as determined using fiber photometry to measure orexin neuron population activities in freely moving mice. The inhibition of orexin neuron activity recovered within seconds of stopping contact with the food. In the present study, c-Fos was used as a marker of the activation of orexin neurons for comparisons between rats that received different foods. Both appetitive and consummatory processes of feeding behaviors contributed to our observations of c-Fos expression, which might also link to various feeding-associated physiological responses, including cardiovascular responses.

Orexin neurons are composed of anatomically and functionally heterogeneous subpopulations. Those involved in reward seeking for food, morphine, or cocaine are located in the LH^40^, whereas orexin neurons in the DMH and Pef are linked to the blood pressure phenotype among SHRs, WKY rats, and Wistar rats^31^. Our finding of palatable-food consumption-associated overactive orexin neurons in the DMH of SHRs is consistent with Clifford *et al*.’s findings that the DMH is linked to the blood pressure phenotype. In addition, the DMH is believed to be an important structure mediating eating-associated cardiovascular responses, because blockade of the glutamate receptor within the DMH decreased the pressor response to eating without affecting feeding behaviors^45^.

In González *et al*.’s study, which demonstrated decreased activity of orexin neurons during eating, the optical fiber was placed near the Pef and LH area. In Mileykovskiy *et al*.’s study, which demonstrated increased activity of orexin-VTA or orexin-LC projections, the recorded orexin neurons were located in the Pef and DMH. It is worth noting that the time course of eating-associated cardiovascular responses (our observations) was similar to that of eating-associated changes in the activity of orexin neurons observed by Mileykovskiy *et al*.^15^. They both remained at an elevated level during the entire course of food consumption. Therefore, the orexin system may be involved in multiple functions required for feeding behaviors with heterogeneous and integrative orexin circuits.

There are limitations of the research designs. First, fasting, refeeding, food restriction, food palatability or combinations of these factors might contribute to c-Fos expression in the hypothalamus and brainstem. Current observations are not sufficient to elucidate or distinguish the contribution of each specific factor. Therefore, whether the difference in c-Fos expression between experimental groups solely resulted from palatable-food consumption is not known. Even so, no matter what factors it might interact with, food palatability was likely a key factor causing the difference in c-Fos expression because it was the only treatment difference between experimental groups. In addition, although current data showed no effect of TCS-OX2-29 on numbers of c-Fos-positive orexin neurons, the role of OX2R in palatable-food consumption-associated activation of orexin neurons required further study to clarify.

Second, SHRs are hyper-responsive to many stressors and stimuli^46–48^. SHRs may also have a hyper-reactive orexin system based on the findings of more orexin neurons and higher reactivity to exogenous orexins in SHRs^22,31^. Our experimental design included many stress factors or stimuli, such as fasting, refeeding, hunger, and palatable food. In addition, fasting and hunger are likely to change sleep-wake pattern which might further activate orexin neurons. Therefore, the possibility that the responses observed in SHRs might have been attributed to these multiple factors cannot be excluded.

In conclusion, in SHRs, DMH orexin neurons were overactive during the consumption of palatable food and might in turn elicit exaggerated cardiovascular responses via an OX2R-RVLM pathway. This finding extends our current understanding of central orexinergic activity involved in neural control mechanisms of exaggerated cardiovascular reactivity not only to stresses but also to a natural behavior (feeding) with pleasant (sweet) stimuli in SHRs. The present findings suggest that the OX2R may be a likely target for better control of phasic blood pressure changes in hypertensive subjects when expose to specific stimuli. Although we do not know whether other kinds of palatable food can cause similar responses, sucrose consumption should be viewed with greater caution especially in hypertensive subjects for better cardiovascular control.

## Materials and Methods

### Animals

Male SHRs and WKY rats were purchased from BioLASCO Taiwan (Taipei, Taiwan) and housed in a room with a 12-h light/12-h dark cycle and regulated temperature (21.1 ± 0.1 °C) and humidity (68.9% ± 0.3%). Animals were allowed free access to food and water before and after the food consumption experiment. All animal procedures were conducted in accordance with guidelines reviewed and approved by the Institutional Animal Care and Use Committee of Taipei Medical University.

### Implantation of an arterial catheter and lateral ventricular cannula

Experiments were performed using 12- to 16-week-old male SHRs and WKY rats. Each rat was anesthetized with an intraperitoneal (IP) injection of Zoletil 50 (tiletamine 25 mg/kg and zolazepam 25 mg/kg) together with Ilium Xylazil (xylazine 10 mg/kg) and placed on a heat-controlled blanket. A catheter (PU-25, Scientific Commodities, Lake Havasu City, AZ, USA) filled with heparinized saline (l00 U/ml) was placed in the abdominal aorta via the right femoral artery to measure the arterial pressure. The distal end of the arterial catheter was subcutaneously tunneled to the scapular region on the dorsal surface of the rat, exteriorized, and secured to the skin. The rat was allowed to recover for 5 to 7 days prior to the experiments.

For experiments in which an ICV injection was required, a guide cannula was implanted into the right lateral ventricle 5 to 7 days after arterial catheterization. Each rat was anesthetized and placed in a stereotaxic device, and a 23-gauge stainless steel guide cannula was positioned 4.5 mm vertically from the surface of the cortex through a burr hole located stereotaxically 1 mm posterior and 1.5 mm lateral to the bregma. The guide cannula was plugged by a stainless-steel stylet and fixed to the skull with three screws and dental acrylic. The rat was allowed to recover for 5 to 7 days prior to the experiments.

### Measurements of the arterial pressure

After surgery, rats were housed individually. The arterial catheter was connected to a swivel via a tether (Instech Laboratories, Plymouth Meeting, PA, USA) mounted on the top of the cage, allowing free movement of the rat. The arterial catheter was flushed with heparinized saline (100 U/ml) every day. During the experiment, arterial pressure was measured by connecting the arterial catheter to a pressure transducer,the output signals of which were sent to a data acquisition system (Biopac MP-36; Biopac Systems, Santa Barbara, CA, USA). The digitized signals were analyzed with the software, Biopac Student Lab Pro vers. 3.7.3 (Biopac Systems), to obtain the arterial pressure, MAP, and HR. The MAP was calculated as the diastolic pressure plus one-third of the pulse pressure.

The basal MAP and HR were calculated as the mean value of data recorded during a 5-min period right before treatment was applied. The MAP and HR at each specified time point are the mean value of the data recorded during a 1-min period around the time point. Changes in the MAP (ΔMAP) and HR (ΔHR) at each specified time point were obtained by subtracting the basal MAP or HR from values at the indicated time point. Maximal ΔMAP and ΔHR were obtained from the mean value of the data recorded during a 1-min period around the peak response of each treatment.

### Intracerebroventricular injection

After removing the stylet, a 30-gauge stainless-steel tube was inserted into the guide cannula. The lower end of the tube extended 0.1 mm past the tip of the guide cannula. The upper end of the tube was connected to a 10-µl Hamilton syringe (with a polyethylene-10 tube) for injecting the test reagent. Each reagent was dissolved in 3 µl of saline and was injected over a 3-min period. The injection tube was left inside of the guide cannula for an additional 30 s and then replaced with the stylet. At the end of each experiment, the rat was anesthetized and received an ICV injection of Evans blue solution (5%). The rat was sacrificed by overdose of anesthesia, its brain was removed, and the position of the cannula in the lateral ventricle was confirmed by diffusion of the dye throughout the ventricular system.

### Experimental Protocols

#### Eating-associated cardiovascular responses

Rats were familiarized with the sucrose agar (3 g/day as a supplement for 2 days) in their home cage environment before the experiments. All experiments were performed in the home cages in an operating room in the animal center of the Taipei Medical University under red illumination. Rats were fasted for 12 h (from the 2nd hour of the light period to the 2nd hour of the dark period) before the experiment began. On the day of the experiment, fasted rats were moved in their home cages to the operating room and allowed to acclimate there for 1 h. Experiments were conducted during the 2nd to 5th hours of the dark period. Arterial pressure was continuously measured during these 3 h. Before any treatment was applied, the arterial pressure and HR were recorded for at least 1 h. Each rat received a piece of 3-g regular chow (LabDiet 5001, PMI Nutrition, Brentwood, MO, USA) or agar block (containing 10% sucrose). Each rat was tested with both regular chow and sucrose agar on different days with at least 1 day apart in a random sequence. Data from rats that completely consumed the food within 20 min were included.

#### Effects of an OX2R antagonist on eating-associated cardiovascular responses

Both SHRs and WKY rats were randomly divided into five treatment groups. Three groups received an ICV injection of vehicle (saline), TCS-OX2-29 (30 nmol), or TCS-OX2-29 (300 nmol) (Tocris Bioscience, Bristol, UK) followed by consumption of sucrose agar. The remaining two groups received an ICV injection of vehicle or TCS-OX2-29 (300 nmol) followed by consumption of regular chow. After at least 1 h of baseline recording, an ICV injection was given, followed by food presentation 30 min later. After 1 h of further recording, SHRs and WKY rats that had received vehicle plus chow, vehicle plus agar, or TCS-OX2-29 (300 nmol) plus agar were sacrificed for immunohistochemical studies. Two additional groups of SHRs (three rats each group) fasted for 12 h with and without TCS-OX2-29 treatment were served as control experiments.

### Immunohistochemistry

#### Tissue preparation

Rats were anesthetized with an IP injection of Zoletil 50 (tiletamine 25 mg/kg and zolazepam 25 mg/kg) together with Ilium Xylazil (xylazine 10 mg/kg) and transcardially perfused with 50 ml of cold heparinized saline (10 U/ml) followed by cold phosphate-buffered saline (PBS, 0.1 M, pH 7.4), and finally by cold 4% paraformaldehyde in PBS. The brain was removed, post-fixed in the fixative overnight, and then cryoprotected with a 30% sucrose-PBS solution. Four brains from each experimental group were further processed for immunostaining. Each brain was coronally sectioned through the midbrain to yield forebrain and hindbrain portions. The forebrain containing the hypothalamus and the hindbrain containing the RVLM were sectioned into 50-µm-thick coronal sections with a cryostat (Cryotome SME, Shandon, Astmoor, UK). One-in-three series of these brain sections were processed for immunostaining.

#### Immunohistochemical staining of c-Fos in the RVLM

Free-floating sections were treated with 3% H_2_O_2_ in PBS for 5 min, blocked with 10% normal goat serum for 120 min at room temperature, and then incubated with a rabbit anti c-Fos antibody (1:400 dilution; SC-253, Santa Cruz Biotechnology, Santa Cruz, CA, USA) at 4 °C overnight. After several washes with PBS, sections were incubated with biotinylated goat anti-rabbit immunoglobulin G (IgG; BA-1000, Vector Laboratories, Burlingame, CA, USA) at room temperature for 2 h, followed by incubation with avidin-biotin complex (Vectastain ABC Elite kit, Vector Laboratories) for 1 h, and c-Fos-immunoreactive nuclei were visualized by reaction with 3,3′-diaminobenzidine (DAB; DAB substrate kit, SK 4100, Vector Laboratories).

#### Double immunofluorescence staining of c-Fos and orexin A in the hypothalamus

Sections were blocked with 10% normal donkey serum for 120 min at room temperature and then incubated with a goat anti-orexin A antibody (1:300 dilution; SC-8070, Santa Cruz Biotechnology) at 4 °C overnight. After several washes with PBS, sections were incubated with biotinylated donkey anti-goatIgG (AP180B, Merck Millipore, San Diego, CA, USA) at room temperature for 2 h, followed by incubation with Alexa Fluor 488-conjugated streptavidin (S32354, Invitrogen/Thermo Fisher Scientific, Carlsbad, CA, USA) at room temperature for 75 min. c-Fos was then labeled with a rabbit anti c-Fos antibody (1:300 dilution; sc-253, Santa Cruz Biotechnology) at 4 °C overnight and detected with Alexa Fluor 594-conjugated donkey anti-rabbit IgG (A21207, Invitrogen/Thermo Fisher Scientific) at room temperature for 75 min. Immunohistochemical control experiments were performed with omission of the primary antibody. Staining was absent in all control experiments.

### Cell counting

Images of immunostaining in the RVLM and hypothalamus were acquired using the TissueFaxs/Zeiss AxioObserver Z1 Microscope System (TissueGnostics GmbH, Vienna, Austria).

#### c-Fos immunoreactivity in the RVLM

Cells with c-Fos immunoreactivity were bilaterally counted in the RVLM area in all stained brainstem sections between bregma −11.8 and −12.8 mm. The RVLM is defined as the area located caudal to the facial nucleus, rostral to the lateral reticular nucleus, and ventral to the nucleus ambiguus and extending to the ventral medullary surface^49,50^. Regions of interest were recognized according to atlases in *The Rat Brain in Stereotaxic Coordinates*^51^. c-Fos-immunoreactive neurons were determined with a clear round or oval aggregation of fully black or distinctly brown nuclear staining. Data are expressed as the average number of c-Fos-positive cells per brainstem section (50-µm thickness).

#### c-Fos immunoreactivity in orexin neurons

Cells with orexin A and c-Fos immunoreactivity in three sub-regions of the hypothalamus, i.e., the dorsomedial hypothalamus (DMH), perifornical hypothalamus (Pef), and lateral hypothalamic area (LH), were bilaterally counted in all stained hypothalamus sections between bregma −2.0 and −4.0 mm. The three sub-regions were divided by two vertical lines^22,31^. The line separating the DMH from the Pef was located two-thirds of the distance from the edge of the third ventricle to the center of the fornix. The other line, separating the Pef from the LH, was located laterally to the fornix with a distance equal to that from the first line to the center of the fornix. Colocalization of the two fluorescent labels was confirmed on a single layer image of the Z stacks. Cell counting was performed by a person blinded to the experimental groups. Data are expressed as the total number of double-labeled cells in each sub-region per rat.

### Statistical analysis

Data are expressed as mean ± standard error of the mean (SEM). A one-factor repeated-measures analysis of variance (ANOVA) with the Bonferroni post-hoc correction was used to examine the main effects of each treatment (consumption of either regular chow or sucrose agar) on ΔMAP and ΔHR over time, and the interaction effects of different treatments with time. When the interaction was significant, differences in ΔMAP and ΔHR between two treatment groups were determined by Student’s *t*-test (Fig. 2A). Mean imputation was used for a few missing values due to signal interference caused by animal movements. A two-factor ANOVA was used to examine interactions between treatment and species for the maximal ΔMAP and ΔHR and for c-Fos expression. All other data were analyzed using a *t*-test or one-factor ANOVA, followed by Duncan’s multiple-range test for between-group comparisons. Statistical significance was set at *p* < 0.05.

The datasets generated during and/or analysed during the current study are available from the corresponding author on reasonable request.
